# Association Mapping of Seed Coat Color Characteristics for Near-Isogenic Lines of Colored Waxy Maize Using Simple Sequence Repeat Markers

**DOI:** 10.3390/plants13152126

**Published:** 2024-08-01

**Authors:** Tae Hyeon Heo, Hyeon Park, Nam-Wook Kim, Jungeun Cho, Changyeun Mo, Si-Hwan Ryu, Jae-Keun Choi, Ki Jin Park, Kyu Jin Sa, Ju Kyong Lee

**Affiliations:** 1 Department of Applied Plant Sciences, College of Agriculture and Life Sciences, Kangwon National University, Chuncheon 24341, Republic of Korea; taehyun@kangwon.ac.kr (T.H.H.); hyeonpark@kangwon.ac.kr (H.P.); jjejje2000@kangwon.ac.kr (J.C.); 2 Interdisciplinary Program in Smart Agriculture, Kangwon National University, Chuncheon 24341, Republic of Korea; knw407@kangwon.ac.kr (N.-W.K.);; 3 Department of Biosystems Engineering, College of Agriculture and Life Sciences, Kangwon National University, Chuncheon 24341, Republic of Korea; 4 Maize Research Institute, Gangwon State Agricultural Research and Extension Services, Hongcheon 25160, Republic of Korea; shr8921@korea.kr (S.-H.R.); jaekeun@korea.kr (J.-K.C.); kjp@korea.kr (K.J.P.); 5 Department of Crop Science, College of Ecology & Environmental Sciences, Kyungpook National University, Sangju 37224, Republic of Korea; sakyujin@knu.ac.kr

**Keywords:** colored waxy maize, near-isogenic line, population structure, anthocyanin content, seed coat color, association mapping, molecular marker

## Abstract

Waxy maize is mainly cultivated in South Korea for the production of food and snacks, and colored maize with increased anthocyanin content is used in the production of functional foods and medicinal products. Association mapping analysis (AMA) is supported as the preferred method for identifying genetic markers associated with complex traits. Our study aimed to identify molecular markers associated with two anthocyanin content and six seed coat color traits in near-isogenic lines (NILs) of colored waxy maize assessed through AMA. We performed AMA for 285 SSR loci and two anthocyanin content and six seed coat color traits in 10 NILs of colored waxy maize. In the analysis of population structure and cluster formation, the two parental lines (HW3, HW9) of “Mibaek 2ho” variety waxy maize and the 10 NILs were clearly divided into two groups, with each group containing one of the two parental inbred lines. In the AMA, 62 SSR markers were associated with two seed anthocyanin content and six seed coat color traits in the 10 NILs. All the anthocyanin content and seed coat color traits were associated with SSR markers, ranging from 2 to 12 SSR markers per characteristic. The 12 SSR markers were together associated with both of the two anthocyanin content (kuromanin and peonidin) traits. Our current results demonstrate the effectiveness of SSR analysis for the examination of genetic diversity, relationships, and population structure and AMA in 10 NILs of colored waxy maize and the two parental lines of the “Mibaek 2ho” variety waxy maize.

## 1. Introduction

Maize (*Zea mays* L.) is an important staple crop on a global scale and occupies a prominent position among staple crops in the regions of Africa, Asia, the Americas, Europe, and Oceania [1]. In particular, two maize types referred to as normal maize and waxy maize, which are classified based on the starch composition found within the seed endosperm, are widely cultivated and used in East Asia, especially in China and South Korea. The main difference between the two maize types is in the texture and starch content of the seeds. Waxy maize possesses a distinctive endosperm texture, characterized by the exclusive presence of branched-chain starch (amylopectin), which accounts for more than 99% of the total composition. In contrast, the starch composition of normal (non-waxy) maize comprises approximately 75% amylopectin and 25% amylose [2,3,4,5,6]. Normal maize is widely grown around the world and serves a variety of purposes, including food production, livestock feed, industrial materials, and biofuel production. Conversely, waxy maize has a special role and is mainly cultivated for the production of food and snacks in East Asian countries, especially South Korea and China, and some Southeast Asian countries such as Vietnam, Laos, Myanmar, and Thailand [4,5,6,7].

Recently, in South Korea, consumer demand for the development of high-quality, highly functional crops and an emphasis on quality over quantity is increasing against the backdrop of an increased national income and heightened health awareness. As a consequence, current maize breeding efforts are also focused on creating functional maize varieties that are enriched with significant amounts of bioactive compounds. Among the spectrum of functional ingredients, it is notable that previous studies have identified anthocyanin pigments as versatile ingredients with various beneficial properties. These include their role as natural colorants [8], their antioxidant potential [9], their utility in the prevention of cardiovascular diseases [10], and their effectiveness as anticancer agents [11].

Therefore, in recognition of the significance and versatility of anthocyanin pigments, the Maize Experiment Station of the Gangwon State Agricultural Research and Extension Services in South Korea undertook in 2011 the development and application of a maize variety named “Colored maize No. 1”, which was specifically engineered to contain high levels of anthocyanin pigments (KOREA SEED & VARIETY SERVICE, http://www.seed.go.kr/, accessed on 1 January 2021, application 2011-131). The creation of such colored maize varieties, enriched with increased anthocyanin content, holds great promise for the advancement of functional foods, medicinal products, and food materials by capitalizing on the valuable properties associated with these pigments. Additionally, waxy maize consumption has increased in South Korea in recent years as consumption patterns have changed from traditional rice-based diets to Western meat and bread-based diets. The Maize Research Institute, under the auspices of the Gangwon State Agricultural Research and Extension Services, has collected and preserved large-scale genetic resources of maize inbred lines and varieties of varying origins from local farmers in South Korea and research institutes and markets in other countries, including China, USA, Vietnam, Cambodia, and Thailand. Currently, the Maize Research Institute’s breeding research team is conducting breeding research with the aim of creating functional waxy maize varieties with distinctive seed coat colors in order to meet the diverse tastes of consumers. As part of this research, the research team is developing functional colored waxy maize varieties through a meticulous backcrossing process, involving “colored normal maize” as the donor parent and “waxy maize” of the parental lines (HW3 and HW9) of the “Mibaek 2ho” variety as the recurrent parent. As a result, through more than 7 years of backcrossing and line breeding, 10 near-isogenic lines (NILs) with various seed coat colors have been bred by the breeding research team of the Maize Research Institute of the Gangwon State Agricultural Research and Extension Services [7]. In particular, the “Mibaek 2ho” variety of waxy maize is the most popular variety in all of South Korea and not just in Gangwon State [7,12]. In this study, in order to develop highly functional waxy corn varieties, we conducted a component analysis of anthocyanin pigments in the seeds of 10 NILs that were used for seed coat color analysis [7]. 

In recent times, technological advances in computer-based image analysis have facilitated its utilization in agricultural phenotyping analysis, especially for assessing seed shape and seed color, plant phenotypic characteristics, and agricultural food quality. A prominent illustration of this application involves the employment of color space conversion as a prevalent pixel preprocessing technique for evaluating food quality. RGB, HSV, and CIELAB stand out as the predominant spatial color models within the realm of food computer vision, as reported by Leon et al. (2006) [13] and Dana and Ivo (2008) [14]. The typical configuration of such systems encompasses five fundamental components, namely an illuminant, a digital camera, an image capture board (such as a frame grabber or digitizer), and the requisite computer hardware and software for image processing purposes, as explained by Quevedo et al. (2010) [15]. This computer hardware and software system can be used effectively for a more scientific evaluation of the seed coat color of colored maize inbred lines. Furthermore, the field of genetic breeding research has made significant advances recently with the adoption of molecular marker-based techniques. In particular, microsatellites or simple sequence repeats (SSRs) have emerged as valuable co-dominant markers and are widely regarded as one of the most suitable tools for evaluating genetic diversity (GD), deciphering genetic relationships, and assessing population structure, primarily because of their reliability, reproducibility, and discriminative capabilities [4,7,16,17,18]. As a result, SSRs have gained extensive utilization in maize breeding research, serving various purposes such as improving and developing new maize varieties, strategic planning of crosses for hybrid or inbred line development, categorizing lines into heterotic groups, safeguarding plant germplasm, and identifying molecular markers linked to valuable morphological traits [4,7,17,18,19,20,21,22].

Uncovering the genetic basis of beneficial agronomic traits suitable for targeted inclusion in plant breeding initiatives is a pivotal contribution to the scientific pursuit of advancing crop enhancement. In recent times, the field of maize breeding has experienced significant transformation propelled by swift technological progress in sequencing and genotyping, encompassing genome editing and doubled haploid technology. These advances are complemented by strides in data sciences and the evolution of innovative breeding methodologies that leverage genomic information [23]. There are two ways to identify genomic regions associated with important traits in crop genetic breeding studies. The first approach, known as quantitative trait loci (QTL) mapping, relies on the results of controlled crosses between two parent lines that exhibit related and contrasting phenotypes and genotypes within backcross populations (BC), F_2_ populations, recombinant inbred lines (RILs), doubled haploid (DH) populations, and near-isogenic line (NIL) populations [4,7,17,18,24]. The second method, referred to as association mapping analysis (AMA), uses linkage disequilibrium (LD) between DNA-based molecular markers and agronomic traits of interest [25,26,27,28,29]. Currently, many breeding populations, such as BC, F_2_, DH, RILs, and NILs, are used for analysis of association mapping in maize breeding research. In recent times, AMA has gained significant attention as the preferred approach to identify genetic loci associated with the inheritance of quantitative or qualitative traits in many crop species [7,22,26,28,29,30,31,32,33]. In its most basic expression, this technique involves the identification of molecular markers that exhibit significant differences in allele frequencies between individuals exhibiting a particular phenotype and a reference group of unrelated control individuals [25,33].

Therefore, in our study, we used GD, genetic relationships, population structure, and AMA of 285 SSR markers along with two anthocyanin content and six seed coat color characteristics among 10 NILs and two parental lines (HW3, HW9) of “Mibaek 2ho” variety waxy maize. Our study aimed to identify SSR markers associated with intriguing characteristics of two anthocyanin contents and six seed coat colors in seeds of the 10 NILs of colored waxy maize. The results of this study are expected to provide valuable information that can be used in future waxy corn breeding programs to develop functional colored waxy maize varieties.

## 2. Results

### 2.1. Anthocyanin Content and Seed Coat Color Analysis and Correlation Analysis

The variations in two anthocyanin contents and six seed coat traits measured in the 10 NILs are summarized in Table 1. Anthocyanin content analysis showed that the kuromanin (cyanidin 3-O-glucoside chloride) content of the 10 NILs was 3.18 ± 5.66 ppm, ranging from 0.175 to 19.5 ppm, and the peonidin (peonidin-3-glucoside) content was 1.21 ± 2.07 ppm, ranging from 0.016 to 7.34 ppm. Meanwhile, the variations in the six seed coat traits of the 10 NILs showed that the average R value was 72.9 ± 37.2 degrees, ranging from 17.9 to 124.7 degrees. The average V value was 72.9 ± 37.1 degrees, ranging from 17.9 to 124.7 degrees. The average L* value was 40.5 ± 27.1 degrees, and ranged from 6.91 to 86.2 degrees. The average a* value ranged from 132.6 to 163.7 degrees, with an average of 150.4 ± 10.0 degrees. The average b* value ranged from 129.2 to 152.8 degrees, with an average of 140.4 ± 7.07 degrees (Table 1). 

We evaluated correlation coefficients among the two anthocyanin contents and six seed coat color traits in the 10 NILs (Table 2). Correlation analysis was performed to confirm genetic relationships between the two anthocyanin contents and six seed coat color traits in the 10 NILs. Among all combinations, the combinations between kuromanin and peonidin (0.988 **), between R and V (1.000 **), between R and L* (0.980 **), between R and a* (0.874 **), between R and b* (0.915 **), between R and 750 nm (0.953 **), between V and L* (0.980 **), between V and a* (0.874 **), between V and 750 nm (0.953 **), between L* and a* (0.760 *), between L* and b* (0.824 **), between L* and 750 nm (0.893 **), between a* and b* (0.974 **), between a* and 750 nm (0.936 **), and between b* and 750 nm (0.920 **) showed comparatively higher positive correlation coefficients than the other combinations, with a significance level of 0.01 or 0.05 (Table 2). Meanwhile, combinations between the two anthocyanin contents and the six seed coat color traits showed negative correlation coefficients, with a significance level of 0.05 (Table 2).

### 2.2. Genetic Diversity and Relationships and Population Structure among 10 NILs of Colored Waxy Maize and Two Parental Lines (HW3, HW9) of “Mibaek 2ho” Variety Waxy Maize

A total of 285 SSR loci were confirmed in a total of 801 alleles in the 10 NILs of colored waxy maize and two parental lines (HW3, HW9) of “Mibaek 2ho” variety waxy maize (Table 3 and Table S1). All SSR loci showed polymorphism, and the degree of polymorphism for each chromosome is shown in Table 3. As a result, a total of 26 SSR loci were polymorphic on chromosome 1, generating a total of 76 alleles. The number of alleles per locus varied from 2 (bnlg2241, umc1013, umc1035, umc1446, umc1715, umc1885, and umc2185) to 4 (bnlg1007, umc1082, umc1177, umc1514, and umc2215), with an average of 2.92 alleles per locus. On chromosome 2, a total of 27 SSR loci showed polymorphism, generating a total of 71 alleles. The number of alleles per locus varied from 2 (bnlg1662, dupssr30b, mmc0271, umc1108, umc1227, umc1265, umc1485, umc1541, umc1560, umc2030, umc2079, umc2205, umc2246, and umc2380) to 5 (bnlg1017 and umc1798), with an average of 2.63 alleles per locus. On chromosome 3, a total of 27 SSR loci showed polymorphism, producing a total of 74 alleles. The number of alleles per locus varied from 2 (bnlg1350a, phi102228, umc1027, umc1286, umc1690, umc1773, umc2105, umc2119, umc2266, umc2269, umc2270, and umc2275) to 5 (umc2101), with an average of 2.74 alleles per locus. On chromosome 4, a total of 35 SSR loci showed polymorphism, producing a total of 101 alleles. The number of alleles per locus varied from 2 (phi295450, bnlg1350a, umc1164, umc1175, umc1303, umc1503, umc1649, umc1667, umc1719, umc1899, umc1969, and umc2176) to 5 (umc1702 and umc2137), with an average of 2.89 alleles per locus. On chromosome 5, a total of 24 SSR loci showed polymorphism, producing a total of 65 alleles. The number of alleles per locus varied from 2 (phi024, umc1325, umc1365, umc1389, umc2111, umc2298, umc2301, and umc2400) to 4 (umc1747), with an average of 2.71 alleles per locus. On chromosome 6, a total of 25 SSR loci showed polymorphism, producing a total of 75 alleles. The number of alleles per locus varied from 2 (bnlg1043, phi078, umc1002, umc1314, umc1857, umc2056, umc2309, umc2320, and umc2324) to 5 (bnlg238), with an average of 3.0 alleles per locus. On chromosome 7, a total of 41 SSR loci showed polymorphism, producing a total of 111 alleles. The number of alleles per locus varied from 2 (bnlg1094, bnlg1792, mmc0171, phi116, umc1066, umc1068, umc1125, umc1339, umc1406, umc1409, umc1546, umc1666, umc1718, umc1872, umc1978, umc1986, umc2177, umc2331, and umc2333) to 5 (umc1015), with an average of 2.71 alleles per locus. On chromosome 8, a total of 28 SSR loci showed polymorphism, producing a total of 74 alleles. The number of alleles per locus varied from 2 (bnlg2046, umc1130, umc1457, umc1724, umc1741, umc1858, umc1868, umc1984, umc2075, umc2182, umc2366, umc2395, and umc2503) to 5 (bnlg1194), with an average of 2.64 alleles per locus. On chromosome 9, a total of 25 SSR loci showed polymorphism, producing a total of 67 alleles. The number of alleles per locus varied from 2 (bnlg1525, bnlg244, bnlg619, dupssr6, phi061, umc1492, umc1634, umc1657, umc2099, umc2134, umc2341, and umc2343) to 5 (umc1131), with an average of 2.68 alleles per locus. Finally, on chromosome 10, a total of 27 SSR loci showed polymorphism, producing a total of 87 alleles. The number of alleles per locus varied from 2 (umc1054, umc1239, umc1432, and umc1645) to 6 (umc2172), with an average of 3.22 alleles per locus (Table 3 and Table S1).

The average major allele frequency (MAF) was 0.592 for the ten chromosomes analyzed (Table 3), with a range of 0.333–0.917 for SSR loci (Supplementary Table S1). The average GD was 0.514 for the ten chromosomes analyzed (Table 3), with a range of 0.153–0.778 (Supplementary Table S1). The average polymorphism information content (PIC) value was 0.434 for the ten chromosomes analyzed (Table 3), with a range of 0.410–0.463 (Supplementary Table S1). Furthermore, the GD index across all SSR loci was as follows: GD (0.153 to 0.778), PIC (0.141 to 0.746), and MAF (0.333 to 0.917) (Supplementary Table S1). The GD index information according to the repeat motifs of the 285 SSR loci is shown in Supplementary Table S3. There were some differences in each SSR primer set, but no significant differences were found. Among the most frequently used di-, tri-, and tetra-nucleotide repeats, di-nucleotide repeats had the highest polymorphism index (Supplementary Table S3). Among the 285 SSR loci, the number of alleles per locus varied from 2 (109 SSR primer loci) to 6 (1 SSR locus, umc2172), with an average of 3.0 alleles per locus. Furthermore, the lowest polymorphism index among all SSR repeat motifs was of penta-nucleotide repeats, while the highest polymorphism was hexa-nucleotide and 15 repeat motifs (umc2328). Additionally, the number of SSR primer loci expressing 5 alleles was 10 (including di-, tri-, hexa-nucleotide repeats, and none). Meanwhile, the 109 SSR loci expressing two alleles did not show any specific repeat motifs. 

**Figure 1:**
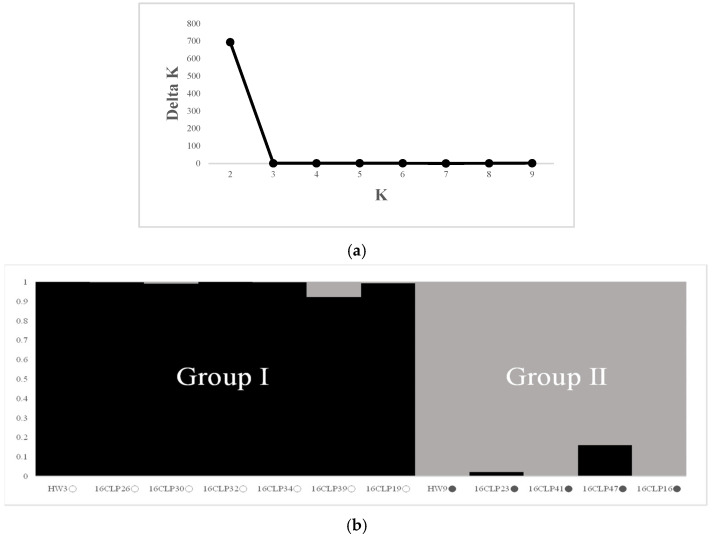
Magnitude of ∆*K* as a function of *K*; the peak value of ∆*K* was at *K* = 2 (**a**). Population structure of 10 NILs and two parental lines (HW3, HW9) of “Mibaek 2ho” variety based on 285 SSR loci for *K* = 2 (**b**). ●: NILs of HW9 BC_3_F_7_, ○: NILs of HW3 BC_3_F_7_.

**Figure 2:**
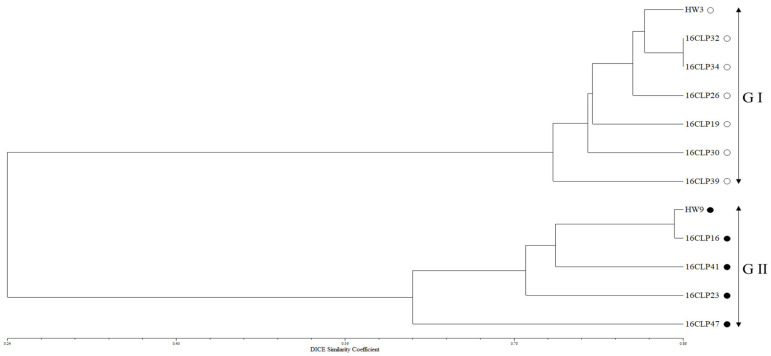
UPGMA dendrogram for the 10 NILs and two parental lines (HW3, HW9) of “Mibaek 2ho” variety waxy maize based on 285 SSR markers.

For the 10 NILs of colored waxy maize and two parental lines (HW3, HW9) of “Mibaek 2ho” variety waxy maize, the highest Δ*K* value was revealed for *K* = 2 (Figure 1a). All the inbred lines studied here were classified into Group I and Group II as follows: seven inbred lines containing HW3 were included in Group I, and five inbred lines containing HW9 were included in Group II (Figure 1b). Furthermore, the dendrogram from the UPGMA analysis is presented in Figure 2. The 10 NILs of colored waxy maize and two parental lines (HW3, HW9) of “Mibaek 2ho” variety waxy maize were clearly classified into two groups with a genetic similarity of 0.62. Group I included seven inbred lines, including HW3, while Group II included five inbred lines, including HW9 (Figure 2). Therefore, the genetic relationships based on UPGMA and STRUCTURE program analysis using SSR markers showed that the 10 NILs and two parent lines (HW3, HW9) of “Mibaek 2ho” variety waxy maize used as recurrent parents in the backcross were clearly divided into the two groups, each group containing one of the two parental inbred lines (HW3, HW9) of “Mibaek 2ho” variety waxy maize.

### 2.3. AMA among 10 NILs Using SSR Markers and Anthocyanin Content and Seed Coat Color Characteristics

From the significant marker–trait association (SMTA) results, 62 SSR markers were found to be associated with the eight seed characteristics in the 10 NILs, using the GLM at a significance level of *p* < 0.05 (Table 4). Among them, for traits related to anthocyanin content, 12 SSR markers (bnlg1017, bnlg238, umc1131, umc1447, umc1490, umc1798, umc1935, umc1946, umc2172, umc2196, umc2255, umc2320) were associated with the kuromanin content trait, and these 12 SSR markers (bnlg1017, bnlg238, umc1131, umc1447, umc1490, umc1798, umc1935, umc1946, umc2172, umc2196, umc2255, umc2320) were also associated with the peonidin content trait. For traits related to seed coat color characteristics, six SSR markers (umc1030, umc1063, umc2122, umc2196, umc2215, umc2255) were associated with both R and V traits, two SSR markers (umc1030, umc2215) were associated with the L* trait, eight SSR markers (bnlg1017, umc1024, umc1030, umc1063, umc1490, umc2122, umc2196, umc2255) were associated with the a* trait, nine SSR markers (bnlg1017, umc1012, umc1030, umc1061, umc1063, umc1490, umc2122, umc2196, umc2255) were associated with the b* trait, and seven SSR markers (umc1030, umc1063, umc1365, umc1490, umc2122, umc2196, umc2255) were associated with the 750 nm trait (Table 4). 

Based on the results, all the anthocyanin content and seed coat color characteristics were associated with SSR markers, ranging from 2 to 12 SSR markers per characteristic. Also, many SSR markers used in our study were together associated with various seed characteristics in the 10 NILs. For example, 12 SSR markers were found to be related to both of the two anthocyanin content traits (kuromanin and peonidin), and six SSR markers (umc1030, umc1063, umc2122, umc2196, umc2215, umc2255) were associated with two seed coat color traits (R and V). Two SSR markers (umc1063 and umc2122) were associated with five seed coat color traits (R, V, a*, b*, 750 nm). One SSR marker (umc1030) was associated with six seed coat color traits (R, V, L*, a*, b*, 750 nm). Also, umc2215 was associated with three seed coat color traits (R, V, L*). Meanwhile, bnlg1017 was associated with both anthocyanin content traits (kuromanin and peonidin) and two seed coat color traits (a*, b*). Two SSR markers (umc2196 and umc2255) were together associated with the two anthocyanin content traits (kuromanin and peonidin) and five seed coat color traits (R, V, a*, b*, 750 nm) (Table 4).

## 3. Discussion

Understanding genetic variation, including GD and relationships, and population structure within breeding materials can provide useful information for breeding programs aimed at developing new maize varieties and valuable maize inbred lines. Specifically, examining the GD, genetic relationships, population structure, and AMA for the 10 NILs of colored waxy maize, which were developed from a backcross breeding program, will provide useful information for identifying molecular markers associated with specific anthocyanin contents and seed coat characteristics and aid in the precise selection of the 10 NILs for cross combinations. Moreover, as consumption of waxy maize increases in South Korea, consumer interest in functional waxy corn is increasing. Therefore, in order to meet this social demand, it is necessary to accurately analyze the genetic characteristics for the 10 NILs of colored waxy maize developed by the Maize Research Institute of the Gangwon State Agricultural Research and Extension Services.

For this investigation, we used a comprehensive set of 285 SSR primer sets in which SSR loci were distributed across the 10 maize chromosomes (ranging from 24 for Ch. 5 to 41 for Ch. 7 per chromosome) to detect genetic profiles in 10 NILs of colored waxy maize and two parental lines (HW3, HW9) of “Mibaek 2ho” variety waxy maize (Table 3 and Table S1). A total of 801 alleles were identified, with an average of 2.81 alleles per locus and 80.1 alleles per chromosome. The average MAF was 0.592 for the ten chromosomes analyzed, with a range of 0.551 (Ch. 1) to 0.642 (Ch. 10) for SSR loci (Table 3). The average GD was 0.514 for the ten chromosomes analyzed, with a range of 0.496 (Ch. 2 and Ch. 10) to 0.545 (Ch. 1) (Table 3). The average PIC value was 0.434, with a range of 0.410 (Ch. 2) to 0.463 (Ch. 1) (Table 3). Although data are not presented in this study, we observed that the 10 NILs exhibited higher GD compared with the two parental lines (HW3, HW9) of “Mibaek 2ho” variety waxy maize (recurrent parent), as previously reported by Kim et al. (2021) [7]. In particular, in the previous study, Kim et al. (2021) [7] performed an analysis of 200 SSR markers; however, in the current study, 285 SSR markers were used in the analysis for a more accurate genetic variability study of the 10 NILs of colored waxy maize. Our findings suggest that the alleles derived from the “Colored maize No. 1” variety, employed as the donor parent, play a more substantial role in enhancing the genetic variation of the 10 NILs compared with the two parental lines (HW3, HW9) of “Mibaek 2ho” variety waxy maize, which is similar to the findings of the previous report of Kim et al. (2021) [7]. Therefore, the results of DNA profiling analysis obtained using the 285 SSR primer sets in this study were considered sufficient for analysis of the genetic characteristics of the 10 NILs of colored waxy maize. Furthermore, the analysis of the variation in seed anthocyanin content and seed coat color characteristics of the 10 NILs showed statistically significant differences in eight seed characteristics used in our study. According to the results of the analysis of correlation coefficients among the two anthocyanin components and six seed coat color traits in the 10 NILs (Table 2), the combinations, particularly between kuromanin and peonidin (0.988 **), R and V (1.000 **), R and L* (0.980 **), R and b* (0.915 **), R and 750 nm (0.953 **), V and L* (0.980 **), V and a* (0.874 **), V and 750 nm (0.953 **), L* and a* (0.760 *), L* and b* (0.824 **), L* and 750 nm (0.893 **), a* and b* (0.974 **), a* and 750 nm (0.936 **), and b* and 750 nm (0.920 **), showed comparatively higher positive correlation coefficients. In contrast, the analysis between combinations of the two anthocyanin components and the six seed coat traits showed negative correlation coefficients, with a significance level of 0.01 (Table 2). These results show a very high positive correlation between seed coat color-related traits, but a negative correlation between the two seed anthocyanin contents and six seed coat traits in the 10 NILs of colored waxy maize. In particular, these results show a very high correlation between seed coat color-related traits and also between seed coat color and anthocyanin content traits in the 10 NILs used in our study. Therefore, these results indicate that these NILs of colored waxy maize could be optimal breeding materials for AMA using SSR markers.

**Figure 3:**
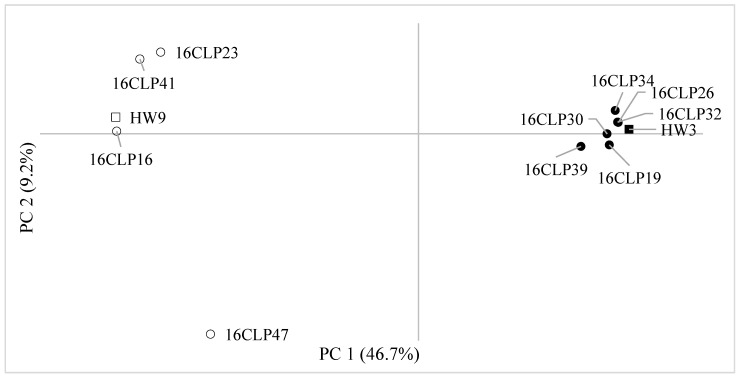
Scatter plot of the PCA for the 10 NILs and two parental lines (HW3, HW9) using 285 SSR markers (●: NILs of HW3 BC_3_F_7_, ○: NILs of HW9 BC_3_F_7_, ■: parental line HW3, □: parental line HW9).

In recent times, different NIL populations have been developed in breeding programs and have been applied widely for thorough examinations of QTL across different crops such as rice, wheat, and maize. For example, in rice, NILs have been employed to identify the loci controlling panicle architecture [34]. Similarly, NILs have been employed to provide the effects of QTL in wheat [35] and maize [36]. In general, these studies involved the development of NILs to assess the impact of one or more QTL. The accurate assessment of QTL effects, including heterogeneous QTL integrated into NILs, has been recognized as a highly effective strategy for characterizing NILs and identifying their molecular properties. Additionally, NILs can be employed as a resource to initiate projects related to positional cloning and to explore broader questions concerning epistatic interactions, genome organization, and genetic linkage [36]. Although NILs have traditionally been utilized to confirm QTL identified in other mapping populations, Szalma et al. (2007) [21] proposed a novel application, suggesting the use of NILs to concurrently map, validate, and integrate QTL into an adapted elite genetic background. The NIL mapping strategies require generating a set of inbred lines, each retaining a small segment of the donor parent’s genome. Collectively, these NILs are intended to represent a substantial portion, if not of the entire genome, of the donor parent [21,36]. Based on our results, the 10 NILs of colored waxy maize displayed diverse variations and correlations in the eight seed characteristics analyzed in this study (Table 1 and Table 2). This result suggests that the seed materials from the 10 NILs are valuable for examining markers associated with both the two seed anthocyanin contents and the six seed coat color traits in each NIL. Furthermore, we found that the 10 NILs of colored waxy maize and two inbred lines (HW3, HW9) of “Mibaek 2ho” variety waxy maize (recurrent parent) were divided into two groups (Groups I and II) as follows: Group I included six NILs (16CLP19, 16CLP26, 16CLP30, 16CLP32, 16CLP34, 16CLP39) and the HW3 inbred line, and Group II included four NILs (16CLP16, 16CLP23, 16CLP41, 16CLP47) and the HW9 inbred line (Figure 1 and Figure 2). The results showed the same grouping pattern with both the STRUCTURE program and UPGMA analysis, as in a previous report by Kim et al. (2021) [7]. Additionally, in principal component analysis (PCA), the 10 NILs of colored waxy maize and the two parental lines (HW3, HW9) of “Mibaek 2ho” variety waxy maize showed the same separation tendency as observed in the previous two analyses (Figure 3). Therefore, the information on the population structure and genetic relationships of the 10 NILs of colored waxy maize is expected to enhance the strategic selection of NILs for cross combinations in the development of new varieties of functional waxy maize in South Korea.

Meanwhile, to increase breeding efficiency in crop breeding research, it is necessary to identify genes associated with important agronomic traits. In breeding programs, QTL mapping has been a common approach in many crop studies over the past decades for detecting genes influencing significant agronomic traits [4,17,18,24,37]. However, for such QTL mapping analysis, it is essential to construct genetic maps using molecular markers for the F_2_, RIL, and NIL populations. Meanwhile, AMA has recently emerged as an alternative to QTL mapping, contingent on available information about population structure, and linkage disequilibrium (LD) mapping is particularly beneficial for pinpointing markers closely linked to specific QTL in natural populations and germplasm accessions [6,7,22,31,32,38,39]. For effective maize breeding programs, it is important to identify genes governing important agronomic traits. In the current study, we developed 10 NILs of colored waxy maize through backcrossing, to develop functional colored waxy maize varieties featuring various seed coat colors. Differing from the white seed coat color observed in the two parent lines (HW3, HW9) of “Mibaek 2ho” variety waxy maize (recurrent parent), the 10 NILs of colored waxy maize showed diversity in the two seed anthocyanin contents and six seed coat color traits. In general, NILs include only a single introgression per inbred line, increasing the power to detect QTL with small effects. Additionally, because most of the genetic background is the same for all inbred lines, NILs have more limited developmental and growth variation, increasing the homogeneity of growth stages within an experiment. Therefore, in our study, the observed differences in seed anthocyanin contents and seed coat color traits among the 10 NILs can be attributed to allelic differences in the chromosomal regions surrounding the introgressed target locus. As pointed out by Stuber et al. (1999) [40], NILs employing elite inbred lines as recurrent parents, which showcase a superior hybrid performance, could prove effective in capturing phenotypic differences between these recurrent parents and NILs. Furthermore, the experimental design should encompass the density of markers employed, with particular emphasis on whole-genome association studies. Among the factors that influence linkage disequilibrium based on SSR primer sets, linkage is considered to be the main factor [6,41]. In our research, we used 285 SSR primer sets, distributed across 10 maize chromosomes ranging from a minimum of 24 (chromosome 5) to a maximum of 41 (chromosome 7) (refer to Table 3 and Table S1), to perform AMA in 10 NILs, and assessed eight seed characteristics, that is, two seed anthocyanin contents and six seed coat color traits. Therefore, the association panel using the 10 NILs and 285 SSR primer sets is considered to be suitable for performing AMA to detect molecular markers while controlling for two seed anthocyanin contents and six seed coat color traits.

The AMA results in our study showed that 62 SSR markers in the 10 NILs were associated with two seed anthocyanin contents and six seed coat color traits. Based on the results, all the anthocyanin content and seed coat color characteristics were associated with SSR markers, ranging from 2 (L* trait) to 12 SSR markers (two anthocyanin content traits) per seed characteristic. In our study, 12 SSR markers (bnlg1017, bnlg238, umc1131, umc1447, umc1490, umc1798, umc1935, umc1946, umc2172, umc2196, umc2255, umc2320) were associated with both of the two anthocyanin content traits (kuromanin and peonidin), while 6 SSR markers (umc1030, umc1063, umc2122, umc2196, umc2215, umc2255) were associated with two seed coat color characteristics (R and V), 2 SSR markers (umc1063 and umc2122) were associated with five seed coat color traits (R, V, a*, b*, 750 nm), 1 SSR marker (umc1030) was associated with six seed coat color traits (R, V, L*, a*, b*, 750 nm), and 1 SSR marker (umc2215) was associated with three seed coat color traits (R, V, L*) (Table 4). Meanwhile, one SSR marker (bnlg1017) was associated with two anthocyanin content traits (kuromanin and peonidin) and two seed coat color traits (a*, b*). Two SSR markers (umc2196 and umc2255) were associated with two anthocyanin content traits (kuromanin and peonidin) and five seed coat color traits (R, V, a*, b*, 750 nm) (Table 4). 

In particular, in our study, we detected two SSR markers (bnlg1017 and umc1447) associated with two anthocyanin content traits (kuromanin and peonidin). These SSR markers are also related to carotenoid concentration, of the carotenoids zeaxanthin and lutein, as previously reported by Kandianis et al. (2013) [42], Ashokkumar et al. (2020) [43], and Zhang et al. (2023) [44]. Therefore, these two SSR markers (bnlg1017 and umc1447) should be related to precursors of carotenoids and anthocyanins, which are typical pigment substances in plants, and they are expected to be useful in the selection of traits related to seed coat color and anthocyanin components in future breeding research for colored maize or waxy maize. In summary, the examination of the two anthocyanin components and six seed coat color traits provided useful information about the seed characteristics of the 10 NILs of colored waxy maize, and the SSR markers linked to these traits may provide useful information for selection in maize breeding programs. Consequently, these novel SSR markers hold potential utility for further studies using AMA related to seed characteristics in both waxy maize and colored maize varieties.

Recently, AMA has been conducted to identify the characteristics of markers and useful traits in many crop species for marker-assisted selection (MAS) in crop breeding programs. In our study, the identification and confirmation of genetic SSR loci associated with eight seed characteristics of 10 NILs of colored waxy maize is expected to provide a good opportunity for maize breeders to perform quality control through MAS. This research demonstrates the effectiveness of SSR analysis in investigating GD, relationships, and population structure and performing AMA within 10 NILs of colored waxy maize and two parental lines (HW3, HW9) of “Mibaek 2ho” variety waxy maize (recurrent parent). Therefore, the obtained data can help optimize the parental selection of crossing combinations in the 10 NILs and aid in the identification of markers for MAS, thereby enhancing seed characteristics in colored waxy maize breeding programs.

## 4. Materials and Methods

### 4.1. Plant Materials and Seed Trait Analysis

The 10 NILs of colored waxy maize and two parental inbred lines (HW3, HW9) of “Mibaek 2ho” variety waxy maize (as the recurrent parent) used in the current experiment are shown in Supplementary Table S1. The Maize Research Institute of the Gangwon State Agricultural Research and Extension Services has developed 10 NILs of colored waxy maize through the following backcross breeding process. These NILs of colored waxy maize were developed from a backcross breeding program between “Colored maize No. 1”, a typical, normal colored maize variety, as the donor parent and two parental inbred lines (HW3, HW9) of “Mibaek 2ho” variety waxy maize as the recurrent parent. Finally, in 2022, the seeds of 10 NILs of the BC_3_F_7_ generation were harvested through backcrossing and self-pollination (single-seed descent method) breeding processes as previously reported by Kim et al. (2021) [7].

Seed coat color analysis of the 10 NILs of colored waxy maize was performed using the following method (Supplementary Figure S1). First, seeds were harvested and subsequently subjected to a drying period of approximately 3 months. Then, in 2023, these dried seeds (30 seeds per NIL) were examined for six distinct seed coat characteristics, namely the red color value of the RGB color space (R), the lightness of the HSV color space (V), the lightness of the CIELAB color space (L*, a*, b*), and hyperspectral image measurement (750 nm) from 400 to 1000 nm (Table 5). Finally, the average color space value of each NIL was measured by extracting the regions of the maize seeds in the image through image processing technology (segmentation). The results of the color evaluation of the seed coats of the 10 NILs of colored waxy maize are summarized in Table 1. 

Furthermore, we measured the contents of two anthocyanins, kuromanin (cyanidin 3-O-glucoside chloride) and peonidin (peonidin-3-glucoside), in the seeds of the 10 NILs of colored waxy maize to compare differences in anthocyanin content in accordance with the six seed coat color types for the 10 NILs. The contents of the kuromanin and peonidin in the 30 seeds of each NIL were determined by high-performance liquid chromatography (HPLC) analysis, which was performed with the Agilent 1260 series system (Agilent Technologies, Santa Clara, CA, USA) with the CAPCELL PAK C18 column (Osaka Soda, Osaka, Japan) as the column used for analysis, as previously described by Oh et al. (2008) [45]. In this study, the statistical analysis of the observed results was performed using the Microsoft Office Excel program (2013), and analysis of variance and correlation analysis of the analysis results were performed using IBM SPSS Statistics version 21.

### 4.2. DNA Extraction and SSR Amplification Analysis

Genomic DNA was extracted from young leaves of 10 NILs of colored waxy maize and two parental inbred lines of “Mibaek 2ho” variety waxy maize using the protocol of the Dellaporta et al. (1983) [46] method with some modifications. In our study, we employed a set of 285 SSR primer sets distributed across 10 maize chromosomes ranging from a minimum of 24 (chromosome 5) to a maximum of 41 (chromosome 7). Among them, 200 SSR primer sets were analyzed in a previous study by Kim et al. (2021) [7], and the remaining 85 SSR primer sets were further analyzed in this study. The 285 SSR primer sets were used for analyses of GD, genetic relationships, population structure, and AMA in the 10 NILs of colored waxy maize and two parental inbred lines (HW3, HW9) of “Mibaek 2ho” variety waxy maize (Supplementary Table S1). Information about the 285 SSR primer sets, including chromosome number, location, and forward and reverse primer sequences, is shown in Supplementary Table S1. This information was obtained from MaizeGDB (http://www.maizegdb.org/, accessed on 1 January 2022). The amplification of the SSRs was carried out in a total reaction volume of 30 µL. This mixture included 20 ng of genomic DNA, 0.2 mM dNTPs, 1× PCR buffer, 0.3 µM of both forward/reverse primers, and 1 unit of Taq Polymerase (Biotools, Madrid, Spain). The polymerase chain reaction (PCR) protocol comprised an initial denaturation step of 5 min at 94 °C, followed by two denaturation cycles of 1 min each at 94 °C, a subsequent annealing step of 1 min at 65 °C, and an extension step of 2 min at 72 °C. Starting from the second cycle, the annealing temperature was incrementally reduced by 1 °C after every second cycle until it reached a final annealing temperature of 55 °C. This final cycle was then repeated 20 times. Upon the completion of all cycles, the final extension step at 72 °C was extended to a duration of 10 min. Following the completion of the PCR reaction, the amplified DNA products were subjected to electrophoresis using a mini vertical electrophoresis system (MGV-202-33, CBS Scientific Company, San Diego, CA, USA). For this procedure, 3 μL of the PCR product was combined with 3 μL of formamide loading dye, consisting of 98% formamide, 0.02% bromophenol blue (BPB), 0.02% xylene C, and 5 mM NaOH. Subsequently, 2 μL of the prepared sample was carefully loaded onto a 6% acrylamide–bisacrylamide gel (19:1) in 0.5X Tris-borate-EDTA (TBE) buffer, and electrophoresis was carried out at 250 V for a duration of 40 to 60 min. The resulting separated DNA fragments were then visualized through staining with ethidium bromide (EtBr) (Supplementary Figure S1).

### 4.3. Data Analysis

The DNA fragments of the 10 NILs of colored waxy maize and two parental inbred lines (HW3, HW9) of “Mibaek 2ho” variety waxy maize amplified for each SSR loci were scored as present (1) or absent (0). The PowerMarker version 3.25 program [47] was used to calculate genetic information such as the number of alleles, MAF, GD, and PIC for the 10 NILs and two parental inbred lines (HW3, HW9). Genetic similarities were calculated between each pair of inbred lines with the Dice similarity index [48]. The similarity matrix was then used to construct a dendrogram using the UPGMA. The dendrogram and PCA constructions were performed using NTSYS-pc.V.2.1 [49]. 

To assess the population structure within the 10 NILs and the two parental lines (HW3 and HW9), we employed the model-based program STRUCTURE 2.3 to confirm the genetic structure [50]. The STRUCTURE program was executed iteratively, conducting five runs for each *K* value ranging from 1 to 10, and employing the admixture model with 100,000 iterations. Subsequently, we computed the average likelihood value, LnP(D), across all runs for each *K*. To determine the most likely *K* value and mitigate potential overestimation of subgroup numbers by STRUCTURE HARVESTER (http://taylor0.biology.ucla.edu/structHarvester/, accessed on 1 January 2023), we employed the ad hoc criterion (Δ*K*) of Evanno et al. (2005) [51]. The run of estimated numbers of subgroups displaying maximum likelihood was then utilized to assign inbred lines with membership probabilities of ≥0.8 to subgroups, while inbred lines with less than 0.8 were assigned to the admixed group (Stich et al. 2005) [20]. AMA was conducted to establish marker–trait associations, employing the program TASSEL 3.0 [52]. This analysis involved the assessment of SMTAs using the Q GLM method, as previously reported by Kim et al. (2021) [7].

## 5. Conclusions

Waxy maize is mainly cultivated in South Korea for the production of food and snacks, and colored maize with increased anthocyanin content is used in the production of functional foods and medicinal products to make use of its valuable properties. We performed AMA for 285 SSR loci, two anthocyanin content traits (kuromanin, peonidin), and six seed coat color traits (R, V, L*, a*, b*, 750 nm) in 10 NILs of colored waxy maize. The 12 SSR markers were together associated with both of the two anthocyanin content traits (kuromanin and peonidin). This research demonstrated the effectiveness of SSR analysis in investigating GD and genetic relationships, population structure, and AMA within the 10 NILs of colored waxy maize and the two parental lines (HW3, HW9) of “Mibaek 2ho” variety waxy maize (recurrent parent). Therefore, the obtained data could help to optimize the parental selection of crossing combinations in the 10 NILs and aid in the identification of markers for MAS, thereby enhancing seed characteristics in colored waxy maize breeding programs.

## Figures and Tables

**Table 1:** Means and standard deviations of two anthocyanin components and six seed coat characteristics in the 10 NILs.

NILs	Kuromanin	Peonidin	R	V	L*	a*	b*	750 nm
16CLP26	1.5	0.895	59.1	59.1	26.3	151.2	139.2	0.394
16CLP30	0.187	0.164	120.8	120.9	86.2	151.9	143.6	0.479
16CLP32	0.305	0.28	88.3	88.3	46.4	159.1	145.7	0.467
16CLP34	1.42	0.355	124.7	124.7	80.0	160.2	146.3	0.498
16CLP39	19.5	7.34	20.8	20. 9	7.43	134.2	129.7	0.278
16CLP19	0.707	0.495	68.7	68.7	32.7	153.9	141.3	0.416
16CLP23	1.09	0.579	66.7	66.7	31.4	153.8	141.4	0.413
16CLP41	0.175	0.016	115.9	115.9	67.2	163.7	152.8	0.467
16CLP47	5.73	1.29	17.9	17.9	6.91	132.6	129.2	0.269
16CLP16	1.20	0.683	45.6	45.6	20.9	143.7	134.9	0.381
Mean	3.18	1.21	72.9	72.9	40.5	150.4	140.4	0.406
Min	0.175	0.016	17.9	17.9	6.91	132.6	129.2	0.269
Max	19.5	7.34	124.7	124.7	86.2	163.7	152.8	0.498
SD	5.66	2.07	37.2	37.1	27.1	10.01	7.07	0.076

Kuromanin: cyanidin 3-O-glucoside chloride, peonidin: peonidin-3-glucoside, R: red color value of RGB color space. V: value of HSV color space, L*: L* value of CIELAB color space, a*: a* value of CIELAB color space, b*: b* value of CIELAB color space, 750 nm: hyperspectral image measurement, Min: minimum value, Max: maximum value, SD: standard deviation value.

**Table 2:** Correlation analysis for two anthocyanin components and six seed coat traits in the 10 NILs.

	Peonidin	R	V	L*	a*	b*	750 nm
Kuromanin	0.988 **	−0.618	−0.618	−0.54	−0.708 *	−0.669 *	−0.737 *
Peonidin		−0.595	−0.595	−0.531	−0.657 *	−0.633 *	−0.687 *
R			1.000 **	0.980 **	0.874 **	0.915 **	0.953 **
V				0.980 **	0.874 **	0.915 **	0.953 **
L*					0.760 *	0.824 **	0.893 **
a*						0.974 **	0.936 **
b*							0.920 **

* Significant at *p* < 0.05, ** significant at *p* < 0.01.

**Table 3:** Genetic diversity index of each chromosome for 285 SSR loci in 10 NILs of colored waxy maize and two parental inbred lines (HW3, HW9) of “Mibaek 2ho” variety waxy maize.

Chromosome	No. of SSR Loci	Total Alleles	Mean of Alleles	MAF	GD	PIC
Ch.1	26	76	2.92	0.551	0.545	0.463
Ch.2	27	71	2.63	0.583	0.496	0.410
Ch.3	27	74	2.74	0.599	0.512	0.427
Ch.4	35	101	2.89	0.574	0.533	0.450
Ch.5	24	65	2.71	0.601	0.513	0.433
Ch.6	25	75	3.00	0.597	0.514	0.445
Ch.7	41	111	2.71	0.583	0.511	0.426
Ch.8	28	74	2.64	0.607	0.499	0.414
Ch.9	25	67	2.68	0.580	0.518	0.428
Ch.10	27	87	3.22	0.642	0.496	0.442
Total	285	801	-	-	-	-
Mean	29	80.1	2.81	0.592	0.514	0.434
Min	24	65	2.63	0.551	0.496	0.410
Max	41	111	3.22	0.642	0.545	0.463

GD: gene diversity, PIC: polymorphism information content, MAF: major allele frequency.

**Table 4:** Information on 285 SSR markers using Q GLM for content analysis of two anthocyanins and six seed coat color characteristics of 10 NILs.

Trait	Marker	Chr.	GLM	Marker R^2^	Trait	Marker	Chr.	GLM	Marker R^2^
Kuromanin	bnlg1017	2	**	0.963	L*	umc1030	3	*	0.506
	bnlg238	6	**	0.946		umc2215	1	*	0.765
	umc1131	9	**	0.948	a*	bnlg1017	2	*	0.870
	umc1447	5	**	0.937		umc1024	2	*	0.489
	umc1490	6	**	0.993		umc1030	3	*	0.612
	umc1798	2	**	0.953		umc1063	6	*	0.593
	umc1935	5	**	0.930		umc1490	6	*	0.727
	umc1946	2	**	0.937		umc2122	10	*	0.617
	umc2172	10	*	0.949		umc2196	6	**	0.804
	umc2196	6	**	0.962		umc2255	3	**	0.723
	umc2255	3	*	0.457	b*	bnlg1017	2	*	0.896
	umc2320	6	*	0.415		umc1012	3	*	0.582
Peonidin	bnlg1017	2	**	0.988		umc1030	3	*	0.575
	bnlg238	6	**	0.973		umc1061	10	*	0.590
	umc1131	9	**	0.974		umc1063	6	*	0.583
	umc1447	5	**	0.973		umc1490	6	*	0.601
	umc1490	6	**	0.987		umc2122	10	*	0.608
	umc1798	2	**	0.988		umc2196	6	*	0.721
	umc1935	5	**	0.973		umc2255	3	*	0.621
	umc1946	2	**	0.973	750 nm	umc1030	3	*	0.553
	umc2172	10	**	0.987		umc1063	6	*	0.591
	umc2196	6	**	0.984		umc1365	5	*	0.409
	umc2255	3	*	0.473		umc1490	6	**	0.769
	umc2320	6	*	0.405		umc2122	10	*	0.582
R	umc1030	3	*	0.568		umc2196	6	*	0.727
	umc1063	6	*	0.598		umc2255	3	*	0.597
	umc2122	10	*	0.591					
	umc2196	6	*	0.628					
	umc2215	1	*	0.732					
	umc2255	3	*	0.516					
V	umc1030	3	*	0.568					
	umc1063	6	*	0.598					
	umc2122	10	*	0.591					
	umc2196	6	*	0.628					
	umc2215	1	*	0.732					
	umc2255	3	*	0.516					

* Significant at *p* < 0.05, ** significant at *p* < 0.01.

**Table 5:** Characteristics used in the analysis of the anthocyanin contents and seed coat traits among the 10 NILs of colored waxy maize.

Abbreviation	Character	When/How Measured	Category
Kuromanin	Cyanidin 3-O-glucoside chloride	after harvest	ppm (μg/mL)
Peonidin	Peonidin-3-glucoside	after harvest	ppm (μg/mL)
R	Red color value of RGB color space	after harvest	Red color degree (0~255)
V	Value of HSV color space	after harvest	Darkness–lightness (0~255)
L*	L* value of CIELAB color space	after harvest	Darkness–lightness (0~255)
a*	a* value of CIELAB color space	after harvest	Greenness–redness (0~255)
b*	b* value of CIELAB color space	after harvest	Blueness–yellowness (0~255)
750 nm	Hyperspectral image measurement	after harvest	Visible light (400~1000 nm region)

## Data Availability

All data generated or analyzed during this study are included in this published article and its Supplementary Materials.

## Supplementary Materials

The following supporting information can be downloaded at https://www.mdpi.com/article/10.3390/plants13152126/s1, Table S1. Characteristics of 285 SSR loci, including marker name and chromosomal location, allele number, major allele frequency (MAF), genetic diversity (GD), and polymorphism information content (PIC) in the 10 NILs and two parental lines of “Mibaek 2ho” variety. Table S2. Near-isogenic lines (NILs) of colored waxy maize and two parental lines (HW3, HW9) of “Mibaek 2ho” variety waxy maize were used in this study. Table S3. Genetic diversity index information according to the repeat motif of 285 SSR primer sets used in the analysis of 10 NILs and two parental lines. Figure S1. The seed coat color of two parental lines and 10 NILs using a DSLR camera (a), and electrophoresis photos using SSR primer of 10 NILs and two parental lines (b) (●: NILs of HW9 BC_3_F_7_, ○: NILs of HW3 BC_3_F_7_).
