# Supplementary material for: Association Mapping of Seed Coat Color Characteristics for Near-Isogenic Lines of Colored Waxy Maize Using Simple Sequence Repeat Markers
Source: Plants (Basel). 2024 Aug 1;13(15):2126. doi: 10.3390/plants13152126 (PMC11313766; doi:10.3390/plants13152126)
Supplement: Supplementary file 1 [file plants-13-02126-s001.zip › Supplementary Figure S1.pdf]

**16CLP26 (○)**	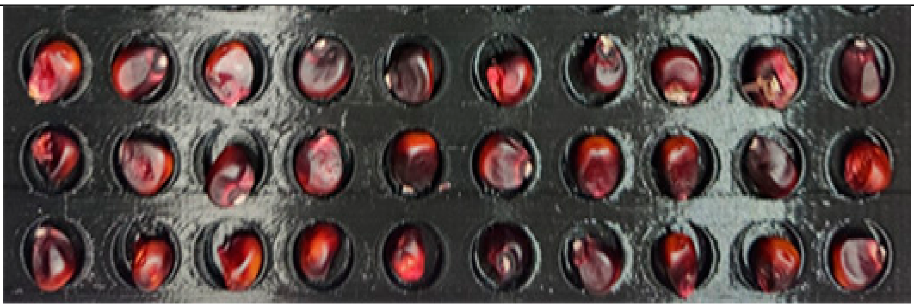
**16CLP30 (○)**	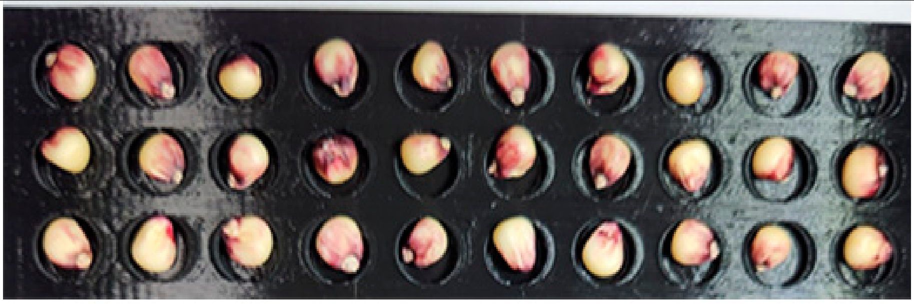
**16CLP32 (○)**	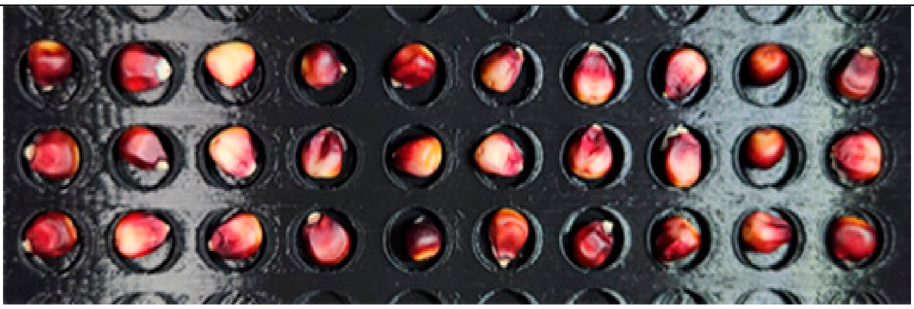
**16CLP34 (○)**	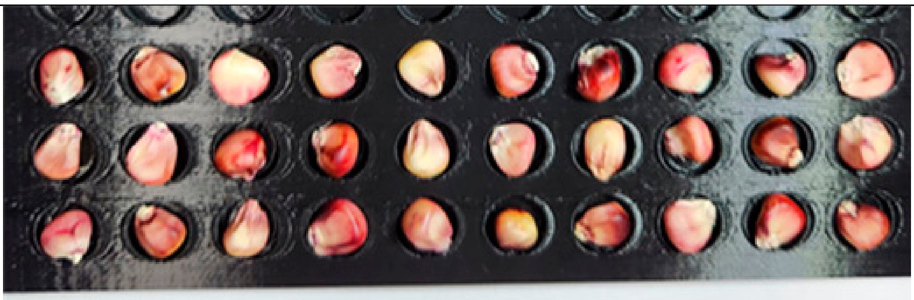
**16CLP39 (○)**	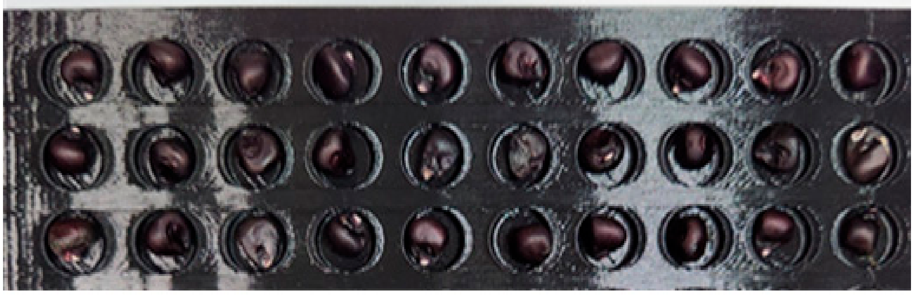

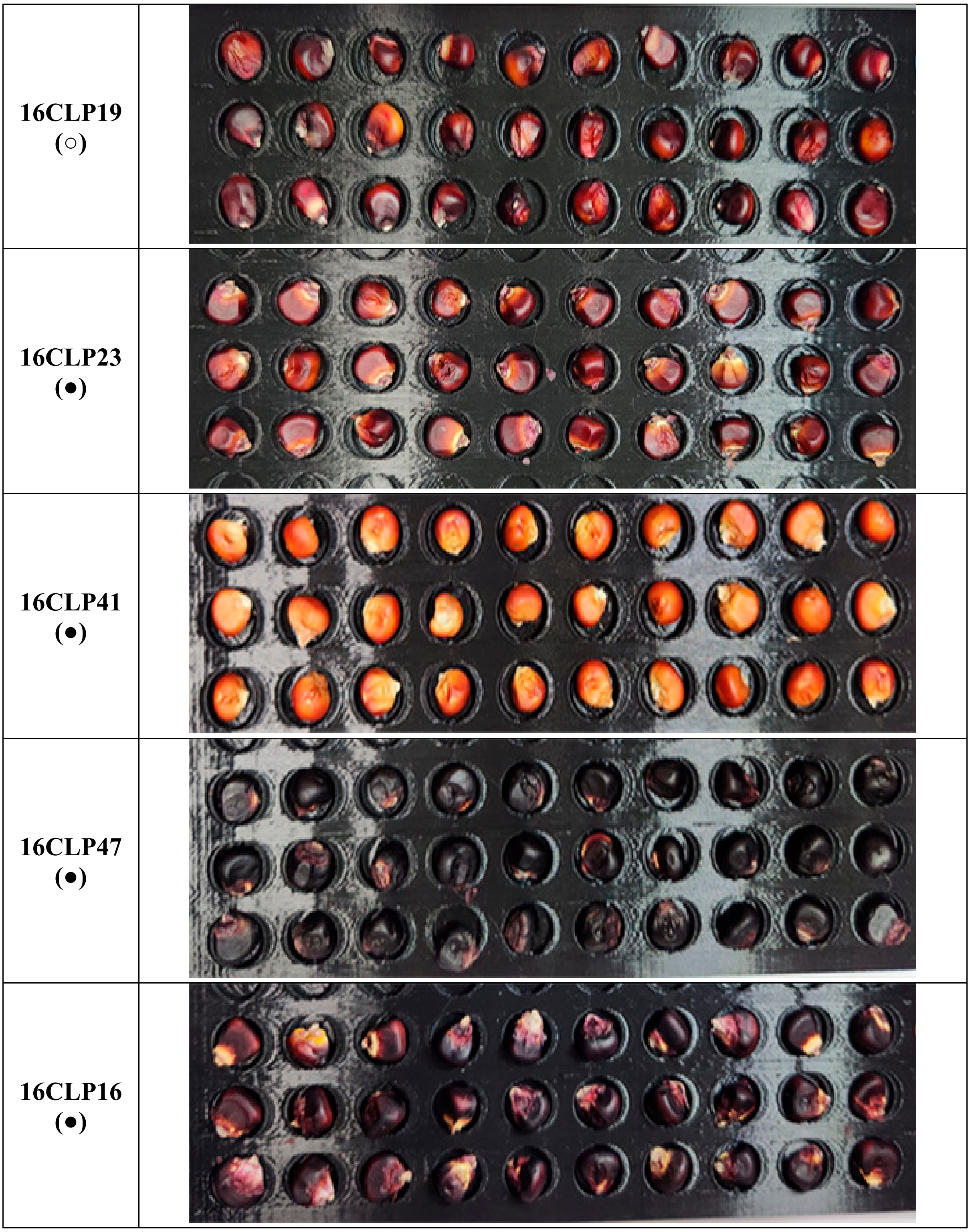

(a)

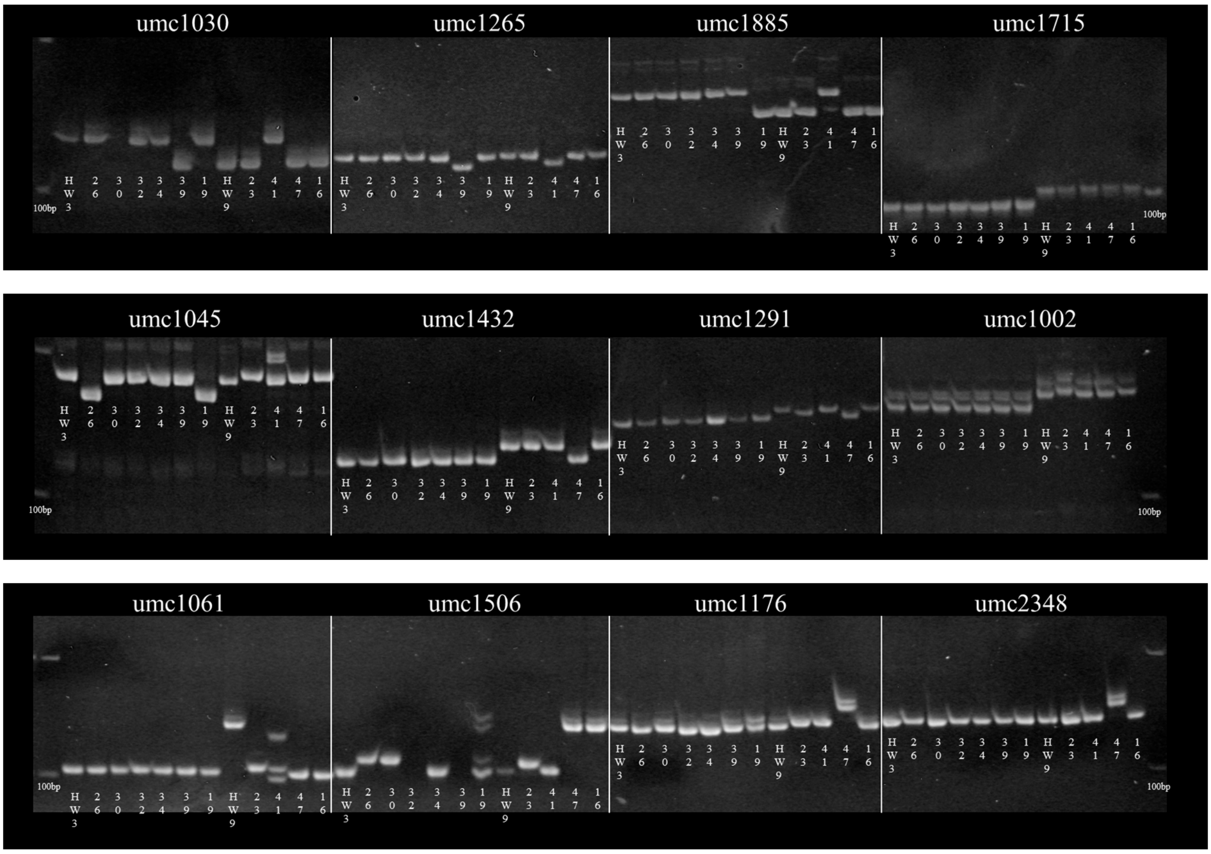

(b)

Supplementary Figure S1. The seed coat color of two parental lines and 10 NILs using a DSLR camera (a), and electrophoresis photos using SSR primer of 10 NILs and two parental lines (b) (●: NILs of HW9 BC_3F_7, ○: NILs of HW3 BC_3F_7).
