# Supplementary material for: Association Mapping of Seed Coat Color Characteristics for Near-Isogenic Lines of Colored Waxy Maize Using Simple Sequence Repeat Markers
Source: Plants (Basel). 2024 Aug 1;13(15):2126. doi: 10.3390/plants13152126 (PMC11313766; doi:10.3390/plants13152126)
Supplement: Supplementary file 1 [file plants-13-02126-s001.zip › Supplementary Table S2.pdf]

Supplementary Table S2. Near isogenic lines (NILs) of colored waxy maize and two parental lines (HW3, HW9) of waxy maize "Mibaek 2ho" variety used in this study

Inbred No.	Name	Backcross
1	HW3	Male of Mibaek 2 variety
2	16CLP26	HW3 BC_3F_7
3	16CLP30	HW3 BC_3F_7
4	16CLP32	HW3 BC_3F_7
5	16CLP34	HW3 BC_3F_7
6	16CLP39	HW3 BC_3F_7
7	16CLP19	HW3 BC_3F_7
8	HW9	Female of Mibaek 2 variety
9	16CLP23	HW9 BC_3F_7
10	16CLP41	HW9 BC_3F_7
11	16CLP47	HW9 BC_3F_7
12	16CLP16	HW9 BC_3F_7

*Data previously published by Kim et al. (2021).
