# Supplementary material for: Association Mapping of Seed Coat Color Characteristics for Near-Isogenic Lines of Colored Waxy Maize Using Simple Sequence Repeat Markers
Source: Plants (Basel). 2024 Aug 1;13(15):2126. doi: 10.3390/plants13152126 (PMC11313766; doi:10.3390/plants13152126)
Supplement: Supplementary file 1 [file plants-13-02126-s001.zip › Supplementary Table S3.pdf]

Supplementary Table S3. Genetic diversity index information according to the repeat motif of 285 SSR primer sets used in the analysis of 10 NILs and two parental lines.

Repeat motif	No. marker	GD	PIC	MAF
di-	118	0.525	0.450	0.583
tri-	79	0.500	0.414	0.600
tetra-	34	0.508	0.422	0.593
penta-	11	0.432	0.378	0.697
hexa-	8	0.552	0.468	0.531
hepta-	1	0.569	0.477	0.500
octa-	1	0.486	0.368	0.583
deca-	1	0.486	0.368	0.583
15 repeat	1	0.569	0.477	0.500
None	31	0.529	0.446	0.578
**Total**	**285**			
**Average**		**0.514**	**0.434**	**0.591**

*None: No repeat motif information in MaizeGDB.
